# Toward Personalized Care and Patient Empowerment and Perspectives on a Personal Health Record in Hemophilia Care: Qualitative Interview Study

**DOI:** 10.2196/48359

**Published:** 2024-09-03

**Authors:** Martijn R Brands, Lotte Haverman, Jelmer J Muis, Mariëtte H E Driessens, Stephan Meijer, Felix J M van der Meer, Marianne de Jong, Johanna G van der Bom, Marjon H Cnossen, Karin Fijnvandraat, Samantha C Gouw

**Affiliations:** 1 Department of Pediatric Hematology, Emma Children’s Hospital Amsterdam UMC location University of Amsterdam Amsterdam Netherlands; 2 Amsterdam Reproduction & Development, Public Health Amsterdam UMC location University of Amsterdam Amsterdam Netherlands; 3 Child and Adolescent Psychiatry & Psychological Care Emma Children’s Hospital Amsterdam UMC location University of Amsterdam Amsterdam Netherlands; 4 Netherlands Hemophilia Patient Society Nijkerk Netherlands; 5 HemoNED Foundation Leiden Netherlands; 6 Department of Thrombosis and Hemostasis Leiden University Medical Center Leiden University Leiden Netherlands; 7 Independent patient representative Utrecht Netherlands; 8 Department of Clinical Epidemiology Leiden University Medical Center Leiden University Leiden Netherlands; 9 Department of Pediatric Hematology and Oncology Sophia Children’s Hospital Erasmus Medical Center Rotterdam Netherlands; 10 Department of Molecular Cellular Hemostasis Sanquin Research and Landsteiner Laboratory Amsterdam Netherlands

**Keywords:** hemophilia, telemedicine, health records, personal, decision-making, shared, patient participation

## Abstract

**Background:**

To enable personalized treatment and shared decision-making in chronic care, relevant health information is collected. However, health information is often fragmented across hospital information systems, digital health apps, and questionnaire portals. This also pertains to hemophilia care, in which scattered information hampers integrated care. We intend to co-design a nationwide digital personal health record (PHR) for patients to help manage their health information. For this, user perspectives are crucial.

**Objective:**

This study aims to assess patients’ and health care providers’ perspectives regarding the use of a PHR in hemophilia care in the Netherlands, required functionalities, and expectations and concerns.

**Methods:**

In this semistructured interview study, 19 pediatric and adult persons with hemophilia, parents, and women with other inherited bleeding disorders, as well as 18 health care providers working within and outside of hemophilia treatment centers, participated. Perspectives of patients and providers were explored separately. To explore requirements, participants were asked to prioritize functionalities.

**Results:**

Participants expected a PHR would increase the transparency of health information, improve patients’ understanding of their illness, and help the coordination of care between health care providers and institutions. Prioritized functionalities included the integration of relevant health information and patient-entered data. Formulated expectations and concerns focused on 4 themes: usability, safety, inclusiveness, and implementation. While patients expressed worries over medicalization (ie, more confrontational reminders of their illness), providers were concerned about an increased workload.

**Conclusions:**

People with hemophilia, their parents, and health care providers welcomed the development of a PHR, as they expected it would result in better coordinated care. Formulated expectations and concerns will contribute to the successful development of a PHR for persons with hemophilia, and ultimately, for all persons with a chronic condition.

## Introduction

### Background

Persons with a chronic health condition, such as the bleeding disorder hemophilia, are encouraged to use eHealth tools to enable personalized treatment and shared decision-making. Hemophilia is an X-linked inherited coagulation factor deficiency of factor VIII (in hemophilia A) or factor IX (in hemophilia B), resulting in an increased bleeding tendency. The severity of hemophilia depends on the residual coagulation factor activity: severe (residual factor activity of <1%), moderate-severe (1%-5%), and mild (6%-40%). Especially in severe hemophilia muscle and joint bleeds are common, leading to painful and disabling joint damage and invalidity. Therefore, lifelong treatment and monitoring is necessary [1,2]. The hallmark of treatment for persons with severe hemophilia consists of replacement therapy with either repetitive prophylactic intravenous infusions of factor concentrate or subcutaneous injections of nonfactor replacement therapy. Preferably, prophylactic treatment is self-administered at home. In case of a bleed, intravenous coagulation factor concentrates are administered, either by people themselves or in the hospital, depending on the severity of a bleed and patients’ expertise. Therefore, people are required to closely self-monitor bleeds and treatment efficacy and seek advice from a hemophilia treatment center if necessary.

Successful treatment demands an active role of the patient and well-developed self-management skills. Self-management is the ability to manage the clinical, psychosocial, and societal aspects of illness and its care, and its impact on life [3,4]. eHealth tools can help to facilitate this active role [5-9]. eHealth tools that are used by persons with hemophilia in the Netherlands include a digital treatment diary to log medication use and bleeding episodes [10], a questionnaire portal to complete patient-reported outcomes measures before follow-up visits [11], and patient portals offered by different health care institutions. However, since each tools has a separate log-in procedure, usability is suboptimal. Similarly, individuals who are treated in various care institutions are required to use multiple patient portals. There is no data exchange between these portals, which results in scattered information. Especially for older individuals with comorbidities, as seen in hemophilia, this hampers integrated care [12,13]. The exchange of information between these eHealth tools, patient portals, and different health care institutions is suboptimal. This results in a loss of information and many requests for the same information, which may negatively impact patient safety [14].

**Figure 1:**
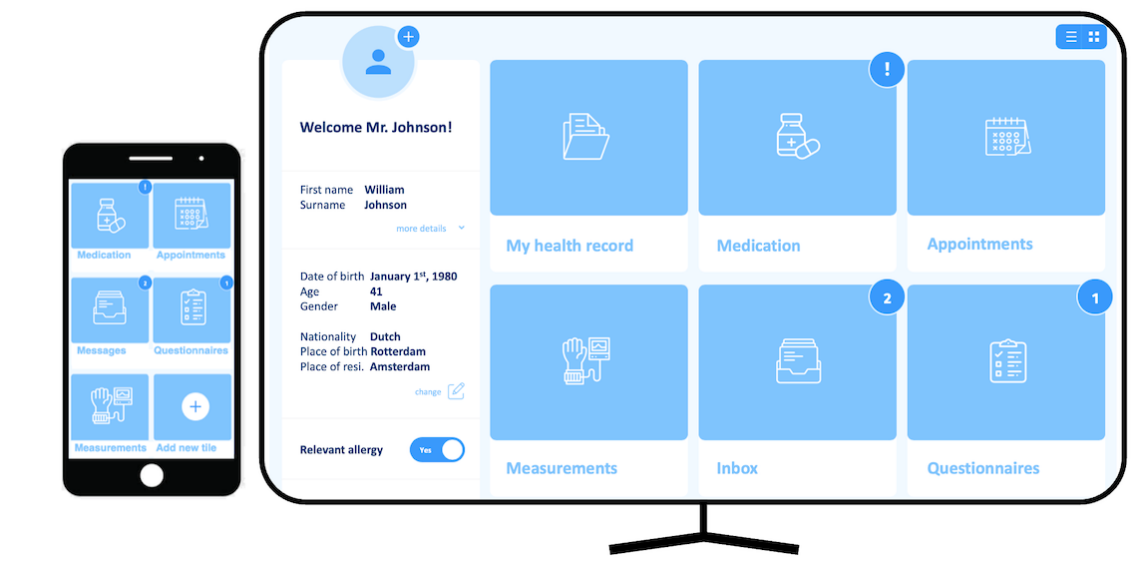
Example of a personal health record, adapted from MedMij.

Personal health records (PHRs) are being developed to resolve these problems and increase patient empowerment [15,16]. In contrast to a patient portal, a PHR’s contents are managed and maintained by individuals, not health care institutions, as illustrated by the definitions presented in Textbox 1. Individuals are able to access and manage their health information and share it with authorized family members or caretakers to help them in managing their care. In addition, people can add self-measurements, such as body weight or data from connected wearables, fill out patient-reported outcomes measures, and complete treatment logs. An example of a PHR is presented in Figure 1. PHRs that are used by people with chronic conditions include PatientsKnowBest (CarePoint) [17] and all certified Dutch PHRs [18]. The Dutch government formulated strict health data exchange guidelines and regulations for PHRs [19]. Therefore, Dutch PHRs will ultimately include integrated medical data from all health care providers involved, regardless of their institution.

Because Dutch PHRs mainly focus on prevalent conditions as hypertension and diabetes, these are not well-equipped in providing for chronic conditions with a high level of self-management, such as hemophilia. Persons with hemophilia may especially benefit from using a PHR, because of the number of health care providers involved in their care, the chronic nature and burden of their condition, and the many self-management skills required from them.

**Definitions.**

**Contents**

- Patient portal: the patient-facing interface of an electronic health record that enables people to view sections of their medical record. This may include access to test results, medication lists, or therapeutic instructions. Health care providers or health care offices determine what health information is accessible for patients. Patient portals often have additional features, such as patient-professional messaging, requesting prescription refills, scheduling appointments, or communicating patient-reported outcomes. By definition, patient portals are “tethered,” which refers to a patient portal’s connection to an electronic health record.
- Personal health record (PHR): a PHR can have similar features as a patient portal. However, the main difference is that contents are managed and maintained by individuals not health care providers. People can access, manage, and share their health information and that of others for whom they are authorized, such as relatives. Health information from different health care institutions may reside in a single patient-managed PHR. Generally, PHRs are not tethered, with the exception of some, including those currently used in the Netherlands.

Note: The definitions are taken from Brands et al [9].

### Objectives

We intend to co-design a nationwide PHR that meets the needs of persons with hemophilia in the Netherlands, their parents, and health care providers. However, their perceptions on a PHR are not yet known. Therefore, our aim was to assess patients’ and health care providers’ perspectives regarding the use of a PHR, required functionalities, as well as expectations and concerns. These insights will help to determine whether a PHR is of value in hemophilia care, and will support its further development.

## Methods

This is a multicenter, semistructured, qualitative interview study, performed in the Netherlands.

### Participants

Study participants were Dutch adolescents and adult persons with hemophilia A and B, parents of young children and adolescents with hemophilia, women with other inherited bleeding disorders, and health care providers working within and outside of hemophilia treatment centers. Adolescents aged 12 to 18 years were interviewed together with a parent. Children <12 years old were represented by the opinions of their parent. Health care providers included: medical specialists, (specialized) hemophilia nurses, allied professionals, and general practitioners (Table 1).

People with hemophilia and parents were recruited through open invitations disseminated by the Dutch Hemophilia Patient Society using their website, email newsletters, and Facebook page. In addition, they were recruited in 2 Dutch hemophilia treatment centers: the Amsterdam UMC and Erasmus MC. We used purposive sampling to include a diverse set of participants. Health care providers were recruited using the direct network of the researchers (MRB and SCG). Inclusion of participants was continued until thematic saturation was achieved, that is, until no new information was introduced in the last 2 interviews. Achieving saturation indicates that additional interviews would not further develop the qualitative theory derived from the data.

**Table 1:** Participant characteristics.

			Patients^a^ (n=19)	Health care providers (n=18)
**Sex, n (%)**				
	Male		14 (74)	2 (13)
	Female		5 (26)	16 (87)
Age (y), median (IQR)			39 (18-65)	—^b^
Work experience (y), median (IQR)			—	16 (8-23)
**Role or function, n (%)**				
	Adult		14 (74)	—
	Adolescent (aged 12-18 y) interviewed together with a parent		2 (10)	—
	Child (aged ≤11 y) represented by the opinion of a parent		3 (16)	—
	Pediatric hematologist or hematologist		—	5 (28)
	Specialized adult or pediatric hemophilia nurse		—	5 (28)
	Pediatric orthopedist		—	1 (6)
	Pediatric physiotherapist or physiotherapist		—	2 (11)
	Infectiologist		—	1 (6)
	Pediatric psychologist		—	1 (6)
	Social worker		—	1 (6)
	General practitioner		—	1 (6)
	Pharmacist		—	1 (6)
**Condition, n (%)**				
		Mild and moderate hemophilia	6 (32)	—
		Severe hemophilia	11 (58)	—
		Von Willebrand disease type 1	1 (5)	—
		Factor VII deficiency	1 (5)	—
**Use of prophylaxis, n (%)**				
		Yes	12 (63)	—
		No	7 (37)	—
**Comorbidities, n (%)**				
		Many	6 (32)	—
		Some	2 (10)	—
		None	11 (58)	—
**Digital expertise^c^, n (%)**				
		Proficient	9 (47)	7 (39)
		Average	6 (32)	6 (33)
		Not proficient	4 (21)	5 (28)
Used teleconsulting, n (%)			5 (26)	10 (56)
Accessed patient portal, n (%)			7 (37)	—
Previous knowledge of PHRs^d^, n (%)			7 (37)	9 (50)

^a^For the 5 parents representing their child, the age and disease characteristics of their child are presented. Among parents, 1 was male.

^b^Not applicable.

^c^A participant’s level of digital expertise, as estimated by the interviewers.

^d^PHR: personal health record.

### Data Collection

Interviews were conducted by MRB (a physician-researcher) and JJM (a physiotherapist-researcher) between September 1, 2020, and February 1, 2021. Interviews were conducted in person or using video conferencing, the latter due to the COVID-19 pandemic. Duration of interviews ranged from 44 to 96 minutes. All interviews were audiotaped and transcribed verbatim. The item topic list, which was used as an interview guide, was based on clinical expertise and experiences and on previous qualitative research conducted by the authors of this study on the quality of hemophilia care [20]. After discussing relevant personal details and current experiences with patient portals, the concept of a PHR was briefly explained using the image presented in Figure 1. Next, participants were asked about their perspective on the use of a PHR in hemophilia care, required functionalities, as well as expectations and concerns.

**Figure 2:**
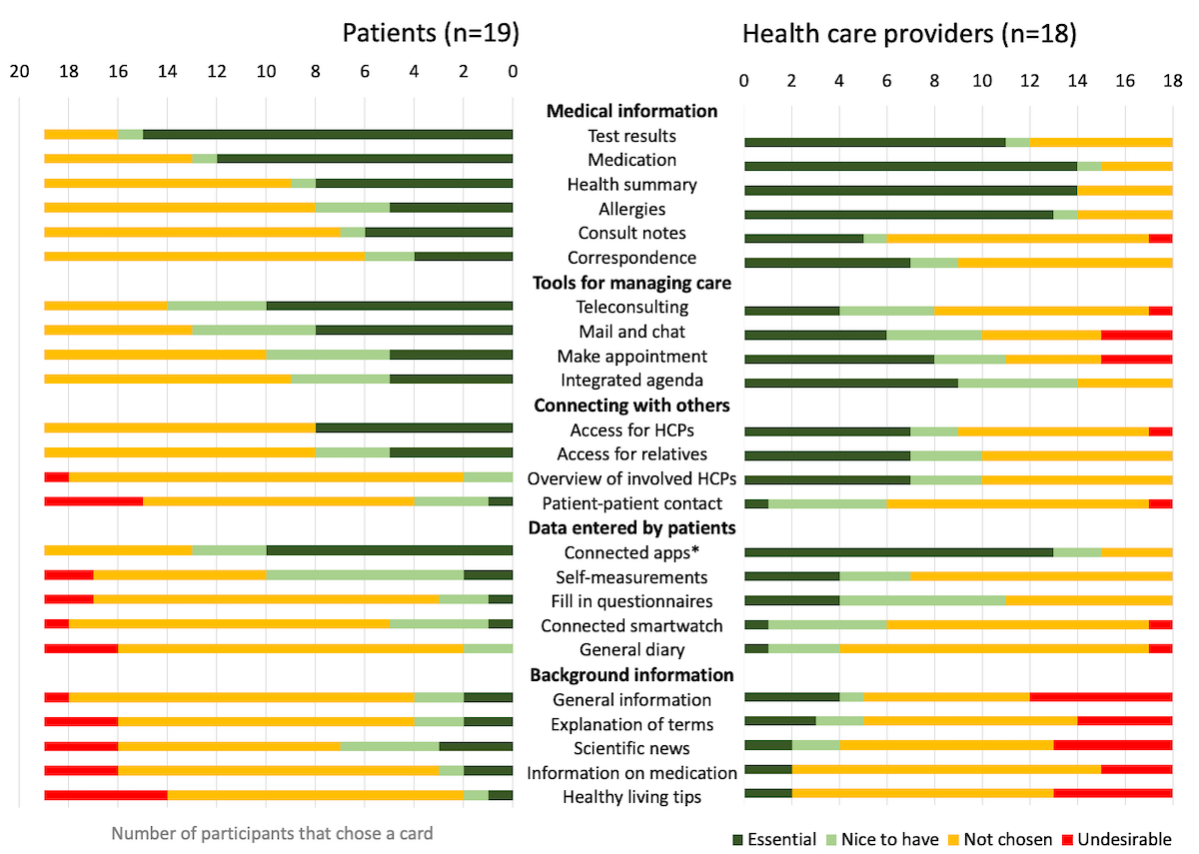
Functionality cards chosen by participants. *Most participants referred to 2 apps: the digital treatment diary app VastePrik and the patient-reported outcome measure portal KLIK. The 25th card, stating, “I do not want a PHR,” is not depicted; this card was chosen by 1 patient. HCP: health care provider; PHR: personal health record.

Regarding required functionalities, participants first openly discussed their ideas. Second, participants were presented a set of 25 cards, each depicting one functionality. Functionalities were split into 5 categories (Figure 2): medical information, tools for managing care, connecting with others, data entered by patients, and background information. The 25th card stated: “I do not want a PHR.” Cards were based on a qualitative study among 31 older adults with a chronic condition and their caretakers [21]. Cards functioned both as prompts and as an exploration of the most required functionalities. Participants were asked to choose the 5 cards which they considered essential. Third, participants chose the functionalities that they considered nice-to-have and those that they considered undesirable. Finally, participants were asked if they had gained new insights regarding the functionalities.

### Data Analysis

In this qualitative study, a directed form of thematic content analysis was used, because elements of our coding scheme were predetermined by our research question. First, using the qualitative software program MAXQDA (VERBI Software), open coding was used to identify themes in interviews. MRB coded all and JM independently coded a third of the interviews. Next, axial coding was used to find connections, and a coding scheme was drafted by MRB and JJM. Thematic discussions were conducted by MRB and JJM, under the supervision of senior researchers LH and SCG.

### Ethical Considerations

The institutional review board of the Amsterdam University Medical Center approved this study (W20_383 # 20.428). All participants signed an informed consent form. Data were deidentified. All participating patients received a gift card worth €20 (US $22) as compensation.

## Results

### Overview

Thematic saturation was reached at a total of 37 participants: 19 (51%) patients and 18 (49%) health care providers. Characteristics are presented in Table 1. Of the 19 patients, 14 (74%) were male. Patients’ median age was 39 (IQR 18-65) years. Furthermore, 11 (58%) had severe hemophilia and 12 (63%) used prophylaxis. In addition, 1 (5%) participant had a factor VII deficiency, and 1 (5%) had von Willebrand disease. Furthermore, 5 (26%) participants were parents of a child with hemophilia. The 18 interviewed health care providers covered all professions involved in the treatment of people with hemophilia. Among them, 7 (39%) worked in adult care, 7 (39%) in pediatric care, and 4 (22%) in both. In total, 16 (89%) were female and median work experience was 16 (IQR 8-23) years.

### Perspectives

#### Overview

In assessing patients’ and health care providers’ perspectives toward a PHR in hemophilia care, three themes were identified: (1) transparency and ease, (2) understanding and control, and (3) coordination and safety. Statements expressed by patients were either patient-oriented or professional-oriented, that is, related to patients or professionals as the primary beneficiaries of PHRs. The same applied to professionals’ statements. Therefore, in exploring themes, and in the matrix in Table S1 in Multimedia Appendix 1, we distinguished patients’ and professionals’ patient-oriented and professional-oriented views. Quotes are presented in Table S2 in Multimedia Appendix 1.

#### Transparency and Ease

Virtually all statements on transparency and ease were patient-oriented. Most patients and health care providers expressed a need for improved access by patients to their health information, by integrating health data into one platform. This is illustrated by quote 1 in Table S3 in Multimedia Appendix 1. Most patients and health care providers expected that improved accessibility would also increase transparency in care activities and treatment considerations (quotes 2 and 3). On a different note, some patients and providers questioned what patients would gain from entering more health data in digital tools, especially if they do not experience any symptoms.

#### Understanding and Control

Patients expressed a desire to gain better, more in-depth understanding of their own health status (quote 4). To achieve this level of understanding, patients said that they require more extensive and detailed health information than is available in the patient portals they were currently using, with additional explanations on what this information means. Such data availability would enable patients to verify if health care providers have understood their symptoms and considerations correctly, and to learn what information is documented (quote 5). In contrast, many patients worried that using a PHR would lead to constant reminders of their illness, and a disturbing confrontation with their illness (quote 6). Several health care providers expressed a similar concern. Finally, many patients expressed a desire to inform health care providers more completely on the impact of illness on their lives, by communicating this through a PHR (quote 7/8).

Health care providers mainly expected that a PHR would improve patients’ insight and self-management skills (quote 9/10). However, many questioned whether sufficient patients would use a PHR, since it requires considerable digital expertise, health literacy, and time investment (quote 11). They observed that many patients currently struggle with the interpretation of medical information in patient portals. Eventually, if more health information would become available in a PHR, this could become even more challenging. And would people who use little medication or have few health appointments find it useful? In contrast, nearly all health care providers would strongly welcome a complete overview of medication and other relevant health data (quote 12). Furthermore, more high-quality information entered by patients would help them to better understand the overall impact of illnesses. However, some health care providers perceived PHRs as a burden that increases their workload and responsibility: would they be expected to verify every data entry (quote 13)?

#### Coordination and Safety

Patients and health care providers elaborated broadly on the final theme: coordination of care and safety. Coordination of care refers to the process of organizing care activities among 2 or more persons involved in a patient’s care, often, including the patient. Consequently, patient- and professional-oriented statements overlap. All participants anticipated a PHR would improve coordination of care (quote14/20). Yet, patients stated that a semileading position in the coordination of their care is already expected from them. Three examples include transferring health information from one health care provider to the next, notifying health care providers in case of particularities, and most importantly, instructing those health care providers who are unfamiliar in treating bleeding disorders what to do in case of emergency situations (quote 16). Yet, most patients feel they have insufficient tools and skills to successfully fulfill this role. They expected that accessing and sharing their medical data would enable them to feel more in control (quote 15). Patients hypothesized it would also reduce preventable mistakes related to allergies, contraindications, and medical history. This view was shared by a minority of health care providers (quote 21).

All participants strongly emphasized the need to improve interprofessional collaborations between health care providers working in different organizations and with different backgrounds (quote 17/22). Still, multiple health care providers questioned whether patients should be able to partially control information flows between health care providers. They feared it is likely that patients will not fully understand all information, and do not always feel proficient to determine who should access their data (quote 18). If patients share all medical information with their care providers, it may overwhelm providers with irrelevant details. However, sharing too little could also result in harmful situations if important information is withheld (quote 19). Finally, some health care providers reasoned that a PHR could result in a more rapid diagnosis of bleeding disorders, especially in female patients, because bleeding symptoms are generally presented to multiple health care providers.

### Required Functionalities

Before presenting functionality cards, participants frequently discussed the following functionalities: an integrated summary of relevant health data and medication, requesting prescription refills, and a complete overview of care appointments and appointment planning.

An overview of prioritized functionality cards is presented in Figure 2. Nearly all participants expected a PHR to contain a complete summary of relevant health information from multiple institutions, including test results, medication, or allergies. Second, integrated access to communication tools, including teleconsulting and chat was considered either essential or nice-to-have by 76% (28/37) of the participants. For functionalities related to appointment making and viewing, this was 78% (29/37). Yet, many health care providers remarked that patients may not have complete freedom in planning their appointments, to maintain some control as a health care provider. Third, 76% (28/37) of participants considered integrated access to commonly used health apps to be either essential or nice-to-have. Participants mainly referred to 2 health apps: a digital treatment diary and a questionnaire portal. They explained that by choosing the card “connected apps,” they implicitly also chose the cards “self-measurements” and “questionnaires.” Participants who did not chose the card “connected apps” were all current nonusers of these apps. Finally, some functionalities were considered less beneficial or unwanted: contacting fellow patients, background information, and scientific updates. One patient chose the card “I do not want a PHR,” because he did not consider himself to be ill and had low health care use. After discussing functionality cards, 3 additional nice-to-have functionalities were brought up: accessing a PHR from abroad, updates on novel treatment options, and explanations of medical terminology.

### Expectations and Concerns

#### Overview

In analyzing patients’ and health care providers’ expectations and concerns, 4 themes were identified: (1) usability, (2) safety, (3) inclusiveness and interpretation, and (4) implementation. The theme matrix is shown in Table S3 in Multimedia Appendix 1 and quotes are presented in Table S4 in Multimedia Appendix 1.

#### Usability

All patients agreed that a PHR must be simple, easy to use, and easily accessible. Many patients stressed on having a low tolerance for crashes and bugs (quote 23). Some expressed usability concerns if PHRs were to have many functionalities. Finally, some said that health care providers working outside of hemophilia treatment centers must also be able to access PHRs, to facilitate shared care.

Health care providers agreed with the necessity of a simple layout (quote 24). They added that an integrated PHR should not only facilitate bleeding disorder care, but all care. Many health care providers stressed that their private work notes must stay private, as is currently the case (quote 25). Finally, health care providers were deeply concerned with an increased workload if they would frequently have to access yet another application with additional health information in the limited time they have (quote 26). Quickly opening PHRs and overseeing all relevant information should meet high usability standards, to not hamper daily practice.

#### Safety

Safety was often mentioned in 2 distinct manners: safety in terms of privacy and in terms of reliable delivery of care. Related to its first meaning, nearly all patients and health care providers expected safety precautions to prevent data breaches (quote 27). As potential culprits, hackers, “big tech” companies, health care insurers, and “big pharma” companies were suggested (quote 30). The latter 2 terms were mostly mentioned by older patients. In addition, many patients insisted to personally authorize every person that may wish to access their PHR.

The second meaning of safety related to the reliable delivery of care. If a PHR were to become an important source of medical information for both patients and health care providers, many stressed it must always be up-to-date and can never go offline (quote 28). Several participants worried that PHRs could delay the delivery of care if patients must approve all information exchanges. Many health care providers worried that patients will not call their health care providers about emergency problems, but use a PHR’s chat function, resulting in a potentially harmful patient and doctor’s delay. Finally, several care providers warned that “bad input” results in “bad output” (quote 29/31). If nonvalidated, inaccurate tools are used to upload self-measurements, treatment plans will be based on incorrect information causing safety risks.

#### Inclusiveness and Interpretation

Many patients and health care providers said that using a PHR should remain optional and may never substitute face-to-face contacts (quote 32). They warned that many patients are likely to experience difficulties using PHRs, including people with low (health) literacy, low digital proficiency, or language barriers (quote 34). Concerns were raised about an increasingly widening “health gap,” by focusing on improving care for those patients who already have good access to care services and leaving behind those who do not. Still, even digital-minded, literate patients may struggle with interpreting and overseeing medical data (quote 33/35). Multiple health care providers referred to “data flooding”—patients who are overwhelmed by the large amount of data, making it impossible for them to determine what is relevant (quote 36). Some suggested this could be resolved by only including essential information in a PHR.

#### Implementation

Many health care providers and some patients mentioned that the complete integration of existing health apps and hospital information systems is essential for the success of PHRs but is challenging (quote 37/39/40). Will vocabulary and abbreviations be exchangeable and understandable for others? Next, many participants stressed it takes “two to tango”—a patient as well as their health care providers need to work with a PHR for it become useful in clinical care. Only if sufficient patients and health care providers use a PHR, it will become part of the routine delivery of care (quote 38).

## Discussion

### Principal Findings

In this study, we assessed patients’ and health care providers’ perspectives regarding the use of a PHR in hemophilia care in the Netherlands, required functionalities, as well as expectations and concerns. Overall, patients and health care providers welcomed the development of a PHR. Both groups recognized the dual effects of using PHRs, either regarding patients or health care providers as its primary beneficiaries. A PHR was generally thought to increase transparency through access to integrated health data, facilitate patients’ understanding of their illness, and improve coordination of care. Requested functionalities were mostly related to the integration of relevant medical information, medication, appointments, communication tools, and data entered by patients. Concerns were mostly related to usability, privacy, patients’ difficulties in managing information flows, variations in patients’ (digital) health literacy, interoperability, and insufficient uptake of PHRs. While patients more often expressed their worries over medicalization (ie, more confrontational reminders of their illness), providers were concerned about their responsibility for all data entered by patients and a potential increase in workload.

### Comparison With Earlier Evidence

The perspectives, required functionalities, and expectations and concerns regarding a PHR or patient portal have not yet been studied among patients with congenital bleeding disorders. More research has been done in other populations.

#### Perspectives

Perspectives toward PHRs and patient portals were assessed by several studies. Perspectives reflect attitudes that define why people consider (or do not consider) using a PHR or patient portal and reveal their motivation and underlying goals. A Flemish questionnaire study assessed perspectives toward a patient portal among the general population [22]. Similar to our study, they found that increased transparency, understanding, and shared decision-making were anticipated. Unlike our study, PHRs’ warning functions for deteriorating health were often mentioned. This might be because patients with a congenital bleeding disorder are already well-educated in self-monitoring treatment effects. Three other studies mainly identified improved patient understanding as the most anticipated benefit of PHRs: a US mixed methods study among older adult patients [23], a US survey among the general population [24], and a German questionnaire study among persons with psoriasis and their health care providers [25]. The latter study also added interprofessional cooperation as an anticipated benefit [25]. Several reviews on patient portals and PHRs revealed a similar trend, in which transparency [26,27] and understanding and control [26-30], and to a lesser extent, coordination, were often stated as motivations for its use [26,30]. Thus, for the greater part, attitudes overlap, although disease-specific differences do occur.

Study participants expected that a PHR will improve the coordination of care. Multiple health care providers questioned whether patients can (partially) control information flows between health care providers due to their limited comprehension of medical concepts. However, patients stated that they are already expected to adopt a semileading position in the coordination of their care. Therefore, with the appropriate support, a PHR could enable patients to better fulfill this semileading role. However, a PHR is not a panacea for all challenges in the delivery of care. A PHR will only improve physician’s delay if health care providers work together more closely. Similarly, by facilitating the exchange of health data, a PHR will not necessarily change the way that health care providers collaborate. Changes in the organization and delivery of care likely need to be made for PHRs to reach their full potential.

Finally, although the term “patient empowerment” was not expressed by patients nor health care providers, this concept occupies a central role in discussing PHRs’ benefits. Empowerment refers to being able to think critically and make autonomous, informed decisions [31-33]. Contrastingly, the concept “self-management” was often mentioned. It can generally be said that, once empowered, patients perform more self-management behavior [4]. In previous studies, the effects of using PHRs and patient portals on self-management ranged from inconclusive [34-38] to beneficial [39-44]. However, few to none of these PHRs truly enabled patients to collect, manage, and share their health information. Therefore, more research is needed to evaluate PHRs while they maturate.

#### Required Functionalities

Most required functionalities identified in this study largely matched those identified in previous studies, although 3 were more prominently listed in other studies: requesting prescription refills [22], viewing clinician contact information [23], and proxy-access for a child or parent [24]. This may be because hemophilia treatment center contact information is already well-known among people with a severe congenital bleeding disorder due to long-term treatment relationships and centralized care. Furthermore, adequate alternatives for requesting prescription refills are already available. Finally, proxy-access was considered essential in a specific subgroup of parents and older adult patients in our study.

#### Expectations and Concerns

In previous studies, the frequently mentioned concerns were usability [16,23,24,26,28,29,34,42-48], privacy and safety [16,23,24,26,27,29,34,42-46,49], inclusiveness [23,27-29,42,43,46,48,50], comprehension of health data [26,29,49], and increased professional workload [16,25,29,48]. Taken together, PHRs are widely expected to be highly user-friendly, understandable, nonobligatory, secure, and inclusive for all patients. Interoperability issues have not previously been reported. However, it is expected that in the rapidly expanding landscape of digital health tools, this concern will become more omnipresent due to the increasing fragmentation of information.

### Strengths and Limitations

A strength of this study is that by identifying both patients’ and health care providers’ perspectives, we were able to assess similarities and differences. Moreover, by using multiple interviewing techniques, such as prompts, participants were stimulated to verbalize their thoughts. Yet, several limitations should be considered. First, by explaining the concept PHR and by using functionality cards, participants may have been influenced. We aimed to limit this risk through the standardization of explanations, and by only presenting cards after we had openly discussed functionalities. Second, by initially including participants through open advertisements and due to the COVID-19 pandemic that forced us to conduct part of the interviews using video conferencing, more digital-minded patients may have been included. Still, by actively including patients from outpatient clinics using purposeful sampling at a later phase, we aimed to minimize such selection. Third, although the gender distribution among the included health care providers represents the current workforce, it is likely that women have different perceptions on a PHR, based on earlier research on gender differences in electronic health record perceptions, although its exact implications remain uncertain [51,52]. Finally, an important limitation is that perspectives and expectations regarding a hypothetical tool are difficult to assess. It may be questioned whether participants truly know what it is that they want and need.

### Implications

The greatest challenge in the implementation of PHRs may prove to be the fragmentation of the digital health landscape and its limited interconnectivity. PHRs may not reach their full potential until different hospital information systems and eHealth tools can be adequately connected. Several national and international initiatives have been established that aim to standardize health information technology [53-56]. Until these programs have yielded effects, we must proactively investigate how to promote inclusiveness of PHRs, and safeguard users’ privacy. This can only be achieved by involving both patients and health care providers in the development of PHRs to acknowledge their different preferences and to motivate both. In doing so, we would advocate focusing on a PHR intended for a broader population of people with chronic conditions and not only for hemophilia, owing to considerable overlap in expectations and requirements. With hemophilia as a representative use case, these insights may aid the development of a PHR valuable for people with any chronic disease with a high level of self-management.

### Conclusions

People with hemophilia, their parents, and health care providers welcomed the development of a PHR in hemophilia care and expressed positive attitudes regarding its use. The benefits of using a PHR on transparency, patients’ understanding of their illness, and the improved coordination of care were widely anticipated. However, concerns regarding usability, privacy, inclusiveness, and interoperability need to be taken into account.

## Appendix

Multimedia Appendix 1 Supplementary tables.

## Abbreviations

PHR: personal health record

## Data Availability

The data sets generated during and analyzed during this study are available from the corresponding author on reasonable request.
