# Supplementary material for: Toward Personalized Care and Patient Empowerment and Perspectives on a Personal Health Record in Hemophilia Care: Qualitative Interview Study
Source: JMIR Hum Factors. 2024 Sep 3;11:e48359. doi: 10.2196/48359 (PMC11408883; doi:10.2196/48359)
Supplement: Multimedia Appendix 1 [file humanfactors_v11i1e48359_app1.docx]

**Table S1** Thematic map of perspectives towards a Personal Health Record in hemophilia care

| **Object** | | **Patient-oriented** | | **Professional-oriented** | |
| --- | --- | --- | --- | --- | --- |
| **Theme\|Quote** | |  |  |  |  |
| **Transparency & ease** | Patient | **✓✓✓**  **✓✓**  **🗶🗶🗶** | Convenient, easy access to all of my health data in one portal.  To access my own data: what is known about me?  Why would I want to have more administration? | **✓✓** | To enable my healthcare providers to access my data. |
|  | HCP | **✓✓**  **🗶** | Increased ease-of-use for patients by offering them one portal in which they can access all of their data and use apps/tools.  What is a patient’s reward for their administration? | **✓** | A PHR might make it easier for us to access data entered by patients information by offering integrated access to all apps/tools. |
| **Understanding & control** | Patient | **✓✓**  **✓✓**  **✓**  **🗶🗶** | To understand what medical data is reported about me.  To check if healthcare providers have understood me correctly.  To do more from my own home, at my own pace.  I don’t want to be reminded of my illness. | **✓✓✓** | To provide a better picture of the impact of illness on my life to my healthcare providers. |
|  | HCP | **✓✓✓**  **✓**  **✓**  **🗶🗶**  **🗶** | To help patients in becoming more involved, with increased disease insight and self-management skills.  To enable shared-decision making by integrating information entered by patients outcomes into one portal, and by better informing patients through the use of PHRs.  To facilitate the complex diagnostic puzzle.  It is only relevant/manageable for a minority of patients.  Patients want to forget their illness. | **✓✓✓**  **✓✓✓**  **🗶**  **🗶** | An up-to-date, correct overview of patient data, especially medication.  To better understand the impact of an illness on a patient’s life.  Are healthcare providers responsible for all data entered in a PHR by patients? How is legal liability and responsibility defined?  Healthcare providers should maintain a certain level of control (e.g. in managing appointments). |
| **Coordination & safety** | Patient | **✓✓✓**  **✓✓✓**  **🗶🗶** | I want to have made my own data available for others (e.g. healthcare providers, relatives) if I am unable to take control (e.g. unconscious, dementia).  To be able to actively send my data to my healthcare providers if needed.  I do not want to be in charge of data flows I do not completely understand. | **✓✓✓**  **✓✓✓**  **✓✓**  **🗶** | To present my data, at a glance, to a (new) healthcare providers, to receive better care.  To improve safety: to prevent mistakes, since my data is ‘out there’ if needed by healthcare providers (e.g. emergency situation).  To help interdisciplinary collaboration among healthcare providers (incl. digital exchange of health data).  It could delay care if patients control data flows |
|  | HCP | **✓✓**  **✓** | It can be a tool for patients to help manage their lives.  To facilitate caretakers in coordinating care. | **✓✓✓**  **✓✓**  **🗶** | To improve the digital exchange of health data between institutions.  To learn which healthcare providers are involved in a patient’s care?  Unsafe if a patient determines data flows |

Themes are depicted in a matrix, in which the interviewee group that expressed a statement (patients or healthcare providers) are shown on the left. Patient-oriented themes and professionals-oriented themes are distinguished: i.e. related to patients or professionals as the primary beneficiaries of PHRs. The number of icons represent the frequency a statement was made, on a scale from 1 (occasionally) to 3 (very often). A **✓** indicates that an attitude expressed by participants was considered to be a positive attitude by the researchers, and a **🗶** a negative attitude.

HCP; healthcare provider

**Table S2.** Patients’ and healthcare providers’ quotes illustrating their perspectives on the use of a Personal Health Record in hemophilia care

| **Transparency & ease** | Patient | Q1  Q2 | “I would absolutely use it. If I could delete all those other apps. Then it’s convenient. Than [a PHR] would become the app that I use to keep track of everything.” – *Male, moderate hemophilia*  “You always have your medical data with you. Simple. You can access it. Always and everywhere.” - *Male, severe hemophilia* |
| --- | --- | --- | --- |
|  | HCP | Q3 | “I would actually consider [a PHR] to function as a patient’s intermediary… or a patient’s gateway to their care. Which shows everything in a well-organized and easily accessible way.” – *Hematologist* |
| **Understanding & control** | Patient | Q4  Q5  Q6  Q7  Q8 | ”It’s reassuring to access my information. Just to read what my doctor has told me, because… for example, when I go to my general practitioner, it is often quite rushed. Since they are so busy. So I like to read, at home, what they said.” – *Male, severe hemophilia*  “Well, it would be nice to know what… what is known about you. Who are you: medically speaking? I think you can learn of lot from that. Especially for someone with hemophilia it might be interesting to learn what is written down and concluded.” - *Male, severe hemophilia*  “You know, I don’t want to know I am ill. If nothing is bothering me, I don’t want to be confronted with it.” – *Male, severe hemophilia*  “Well, I can imagine that in a PHR I would keep track of things that are hard to talk about. Mood… sexuality. […] It would then be easier to discuss, and would be easier for a physician to ask questions about.” – *Female, inherited coagulation disorder*  “To inform. What information do you send to your healthcare provider so he can construct a complete picture of you as a patient, so he can help you in the best way he can.” – *Mother of son with severe hemophilia* |
|  | HCP | Q9  Q10  Q11  Q12  Q13 | “You simply create more possibilities for patients to take control over their lives, and… I believe that is very important. We know that more health knowledge among patients results in better care.” – Psychologist  “A PHR could also provide feedback. Patients can reflect on their health and how it has developed over time.” – Pediatric hematologist  “Users will likely be well-educated, have access to modern technology and have time for this. When you have a busy life, you won’t use this. […] And multiple diseases, yes, that’s also a requirement.” - Infectiologist  “When there is a place where all pills are listed, and which is reliable… Everybody [all HCPs] would log in for that.” – Infectiologist  “I just don’t want to be introduced to such a responsibility. However, I am already put in the position in which people send me information. Simply to make sure that ‘they’ve mentioned it’… And then it is up to me, the doctor, to fix it. Then it’s on my plate. I’m just not sure how this is sorted medico-legally [in the context of a PHR].” – *Orthopedist* |
| **Coordination & safety** | Patient | Q14  Q15  Q16  Q17 | “Maybe, as a mother, you want to have even more control than when you are a patient yourself. […] Because I need to take care of him. You know. And… this information sort of lets me get a grip on everything. [...] I cannot prevent him from bleeding, but I can make sure that when it happens, we are able to help him quickly and help him well.” – *Mother of son with severe hemophilia*  “When I think about why I would use a PHR... Well, so that all healthcare providers, at a glance, can see my son’s health status. What are the risks and dangers?” – *Mother of son with severe hemophilia*  “The fact that they [HCPs] can access your personal health record when you are unconscious, is something I find comforting. When I are unable to take control myself.” – *Male, mild hemophilia*  “The digital exchange of healthcare information is very important. That should be improved upon.” - *Male, severe hemophilia* |
|  |  | Q18 | “But… I’m not sure if I am able to determine whether someone should be able to access my medical file or not.” – *Male, severe hemophilia* |
|  | HCP | Q19  Q20 | “I don’t understand what information every potential health professional needs. For example: a skin problem. Does a skin doctor needs to know about my diet? So… I would sort of be performing the tasks of a general physician. That’s tricky.” – *Male, moderate hemophilia*  “The fact he [a patient] has 14 healthcare providers circling around him… I think to myself: jeez, how can he manage that? If a PHR could help him in doing so? All professionals could then… communicate with him…. and because he can access all of his information and all tools needed to manage his disease.” – *Pediatric hematologist* |
|  |  | Q21  Q22 | “Most importantly, patients can access their data. And healthcare providers can see it too. Because when you are hit by a car in some small village, and are brought to the emergency department of a small-town hospital, they [HCPs] should instantly be alerted about your hemophilia.” – *Specialized nurse*  “Everybody realizes that currently, the exchange of medical information is a mess. It needs to be better. This [a PHR] could help with that.” – *Hematologist* |

HCP; healthcare provider. Pat; patient.

**Table S3.** Thematic map of patient’s and healthcare providers’ expectations and concerns regarding the use of a Personal Health Record (PHR) in hemophilia care

| **Theme\|Quote** | | **Expectations** | | **Concerns** | |
| --- | --- | --- | --- | --- | --- |
| **Usability** | Patient | ✓✓✓  ✓✓  ✓  ✓ | Simple and attractive lay-out  Simple and safe log-in procedure  Modular: possibility to add/remove features  Non-HTC healthcare providers can also access a patient’s PHR | **🗶🗶**  **🗶** | Technical crashes or bugs  Unclear lay-out if there are many functionalities present |
|  | HCP | ✓✓✓  ✓✓✓  ✓ | Very simple lay-out  Limitations in how many HCP notes/letters can be accessed  Usable for hematologic and non-hematologic conditions | **🗶🗶🗶** | Increased workload for HCPs |
| **Safety** | Patient | ✓✓✓  ✓✓✓  ✓✓ | Excellent security  Patients can determine access to their PHR through authorization  Information is always up-to-date | **🗶🗶🗶**  **🗶🗶**  **🗶🗶**  **🗶** | Data breaches (hackers, big pharma, big tech or health insurers)  May cause delays or hinder communication  Problems with accessibility (PHR goes offline)  Bad input = bad output (inaccurate measurements, invalid tools) |
|  | HCP | ✓✓✓  ✓ | Excellent security  Patients may not alter medical data (preserve data reliability) | **🗶🗶🗶**  **🗶🗶🗶**  **🗶🗶** | Data breaches  Bad input = bad output  Patients who use the PHR chat in case of emergency |
| **Inclusiveness & interpretation** | Patient | ✓✓  ✓✓  ✓ | Optionality in using a PHR  PHRs can’t substitute real-life contacts (should be additional)  Patient support desk, e.g. to help decide which HCP should receive what data | **🗶🗶🗶**  **🗶🗶🗶**  **🗶🗶** | Patients cannot fully interpret medical data & discern main/side issues  Difficult to use for some (e.g. elderly, low digital proficiency, low literacy)  Patients cannot always correctly determine which HCP should receive what data |
|  | HCP | ✓✓  ✓ | Only essential data in PHRs to prevent information overload (or available features to filter main/side issues)  Patient support desk, e.g. to help patients what data they must or must not share with HCPs | **🗶🗶🗶**  **🗶🗶🗶**  **🗶🗶🗶**  **🗶🗶**  **🗶** | Certain patient groups may be excluded, resulting in a widened ‘health gap’  Patients cannot fully interpret all data  Patients are flooded with data, but how to decide what is relevant?  Patients cannot determine what HCPs should receive what data  HCPs are flooded by irrelevant patient entered data |
| **Implementation** | Patient | ✓ | Total integration of existing apps and tools in a PHR | **🗶🗶**  **🗶**  **🗶** | Interoperability with hospital information systems and e-Health tools may not always be possible  Can private PHR developers be regulated (beware of the commercialization of healthcare)  If insufficient HCPs and patients use a PHR, it is of no added value |
|  | HCP | ✓✓✓  ✓ | Total integration of data, systems and apps  Should be of added value straight from its launch (no pilot) | **🗶🗶🗶**  **🗶🗶**  **🗶**  **🗶** | Technical challenges: interoperability of health information systems  Sufficient patients *and* HCPs need to use a PHR before it will be successful  Each HCP considers different data relevant; how to personalize?  Can HCPs understand each other’s letters and abbreviations? |

The number of icons represent the frequency a statement was made, on a scale from 1 (occasionally) to 3 (very often). A **✓** indicates that an attitude expressed by participants was considered to be a positive attitude by the researchers, and a **🗶** a negative attitude.

HCP; healthcare provider, HTC; hemophilia treatment center, Pat; patient, PHR; personal health record

**Table S4.** Patients’ and healthcare providers’ quotes illustrating their expectations and concerns regarding the use of a Personal Health Record (PHR) in hemophilia care

| **Usability** | Patient | Q23 | “It will succeed or fail depending on how well it functions. As is the case with all technology. When there is this minor thing that you will find inconvenient, you will stop using it. That’s how it is. You’ll toss it away very quickly.” – *Female, inherited bleeding disorder* |
| --- | --- | --- | --- |
|  | HCP | Q24  Q25  Q26 | “I want it to be very, very easy to use, to be accessible and… almost as if it were an Apple tool.” - Psychologist  “Of course, patients and healthcare providers are partners, but we aren’t completely symbiotic. There is a gap, and we should respect that. Each other’s private domain.” – *Pediatric hematologist*  “I can’t say I am looking forward to it. It is going to be yet another action and yet another thing to discuss.” – *Hematologist* |
| **Safety** | Patient | Q27  Q28    Q29 | “Data is the oil of the 21st century.” – *Male, severe hemophilia*  “The value of this instrument is completely dependent of its maintenance. […] It should always be up-to-date. […] If there would be a delay in uploading a healthcare provider’s latest notes onto a PHR… of days, maybe a week… and if an emergency situations would happen and another healthcare provider looks in a PHR and misses information that might have been essential…” – *Male, severe hemophilia*  “There should be a very thorough selection: which apps will be connected to it? Since many of these apps are not that great. Then you will basically receive bad advice based on bad data.” – *Male, severe hemophilia* |
|  | HCP | Q30  Q31 | “Privacy. That is the biggest threat. That people can access this [a PHR] without the knowledge and permission of a patient.” – *Hematologist*  “Many pedometers are not reliable and valid. This will likely also be the case for… for example, blood pressure monitors.” - *Physiotherapist* |
| **Inclusiveness & interpretation** | Patient | Q32  Q33 | “Care shouldn’t become too numerical. It should always be… healthcare providers shouldn’t think: ‘well, I have seen his PHR, now I have seen it all’, while they haven’t even talked to the patient. Care should remain people work.” – *Mother of son with moderate hemophilia*  “Test results are listed [in available patient portals], but without normal values and explanations. So by itself, these results are not very informative.” – *Mother of son with moderate hemophilia* |
|  | HCP | Q34  Q35 | “Naturally, there will be people who will drop out. Because of their self-management skills, their IT skills, their IQ, socioeconomic status, their character: whether they are disciplined or nonchalant...” – *Pediatric hematologist*  “As a patient, you probably have no clue what type of information [your medical history] contains. Even that suicide attempt when you were 18, which is still listed there when you are 50. For women: all their abortions…” – *Infectiologist* |
|  |  | Q36 | “We should remain vigilant: what information will be entered in one’s personal record. […] For example: in our hospital’s electronic health record, each follow-up note is automatically started with a patient summary. It’s repeated every single time. […] So, in the end, you are left with a gigantic diarrhea of data. You can’t even imagine! You really don’t want to copy all of this onto a PHR.” – *Hematologist* |
| **Implementation** | Patient | Q37  Q38 | “I would use it, if I won’t be needing these other tools anymore. If [a PHR] would have 80% of all functionalities, but you would still need to access a different app now and again… then it would almost be like yet another platform.” – *Female, inherited coagulation disorder*  “There is a critical mass before it’s useful. If only 10% of patients use it, a PHR won’t be that interesting for doctors.” – *Male, severe hemophilia* |
|  | HCP | Q39  Q40 | “Just to indicate that… I am looking at six different screens. You barely see the patient anymore. When I have to check all of that… That is something you have to watch out for: you have to integrate things.” - *Hematologist*  “It should function well right from the start. If you’d release an unfinished product, and functionalities are limited… people will not use it.” – *Hematologist* |

Pat; patient, HCP; healthcare provider
